# A case report and literature review: one case of ceftriaxone sodium-induced reversible gallbladder stone

**DOI:** 10.3389/fmed.2024.1445228

**Published:** 2024-11-27

**Authors:** Changqin Luo, Qingmin He, Xiaocui Yang

**Affiliations:** ^1^Department of Gastroenterology, Ankang Central Hospital, Ankang, Shaanxi, China; ^2^Henan Key Laboratory of Helicobacter Pylori, Microbiota and Gastrointestinal Cancer, Marshall Medical Research Center, Fifth Affiliated Hospital of Zhengzhou University, Zhengzhou, Henan, China

**Keywords:** ceftriaxone sodium, pseudolithiasis, gallbladder reversible stones, ultrasonography, adverse drug reactions

## Abstract

This paper reports a case of pseudolithiasis caused by the use of ceftriaxone sodium. The patient, a 54-year-old female, presented with intermittent abdominal pain and distension for 1 month. Initial ultrasonography showed no abnormalities. However, after receiving ceftriaxone sodium treatment for an upper respiratory tract infection, she developed discomfort in the right upper abdomen. A CT scan subsequently diagnosed her with gallbladder stones. Six weeks after discontinuing the medication, a follow-up examination revealed the disappearance of the stones, confirming the diagnosis of drug-induced pseudolithiasis. Through a literature review, this paper summarizes the mechanism, clinical manifestations, diagnostic methods, and treatment recommendations of ceftriaxone sodium-induced pseudolithiasis, providing a reference for clinical safe medication use.

## 1 Introduction

Ceftriaxone sodium, as a semi-synthetic third-generation injectable cephalosporin antibiotic, has been widely used in international clinical practice. Its unique antibacterial properties are exhibited by its high stability against the broad-spectrum β-lactamase produced by Gram-negative bacilli, especially showing significant antibacterial efficacy against Gram-negative bacilli such as Enterobacter. After being absorbed in the human body, this drug can penetrate tissues effectively and achieve wide distribution in the body. However, with its increasingly widespread clinical use, reports of related adverse drug reactions are also on the rise (1). This article thoroughly analyzes a case of pseudo-gallbladder stones caused by the injection of ceftriaxone sodium, and systematically summarizes the clinical manifestations, formation mechanism, and corresponding treatment measures of pseudo-stones induced by ceftriaxone sodium. It is hoped that this study can provide clinicians with safer medication references and guidance.

## 2 Case presentation

**Figure 1:**
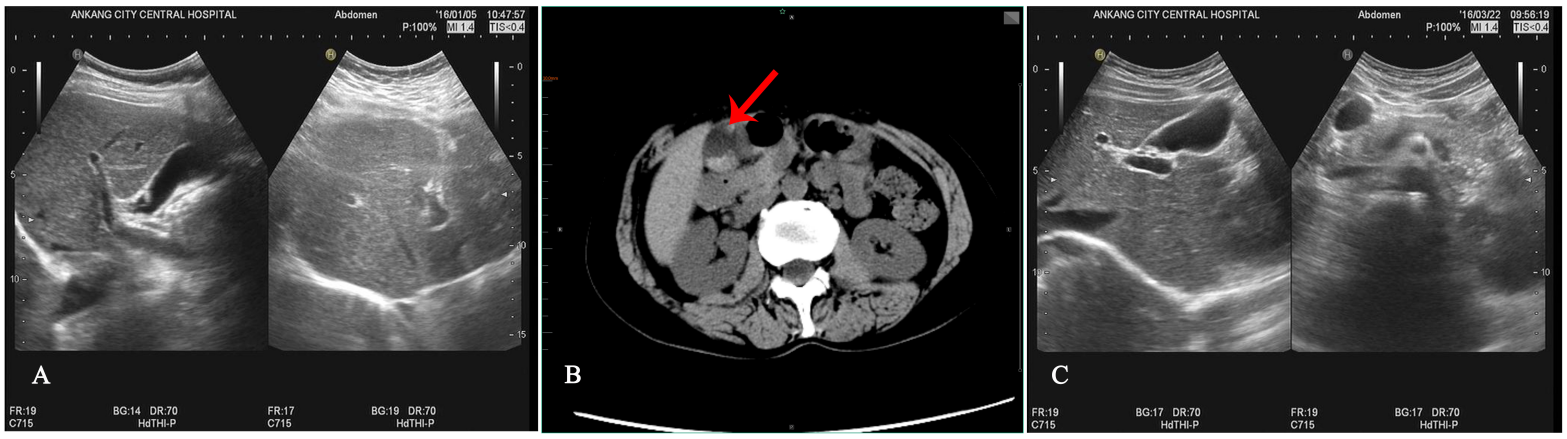
Results of auxiliary examinations. **(A)** Abdominal B-ultrasound before ceftriaxone sodium administration indicates a smooth gallbladder wall with no strong echogenicity in the lumen. **(B)** Abdominal CT after ceftriaxone sodium administration reveals a normal-sized gallbladder with a smooth wall and multiple nodular high-density shadows within. **(C)** Follow-up B-ultrasound 42 days after symptomatic treatment shows the disappearance of gallbladder stones.

The patient is a 54-year-old middle-aged female farmer who presented to the gastroenterology department of our hospital with a 1-month history of intermittent abdominal pain and distension. Upon inquiry, the patient denied any prior history of hepatobiliary calculi, chronic diseases such as diabetes and hypertension, any family history of similar illnesses, and any history of smoking, alcohol consumption, or unhealthy lifestyle habits. Initial physical examination revealed tenderness below the xiphoid process without rebound tenderness, and no other significant abnormalities were observed. To further diagnose, an upper abdominal ultrasonography was performed, which showed no obvious abnormalities in the liver, gallbladder, pancreas, and spleen (Figure 1A). Meanwhile, gastroscopy revealed superficial gastritis (in the antral region), and the C14 breath test was negative. Based on the patient's symptoms, oral rabeprazole was administered for treatment. After treatment, the patient's abdominal distension symptoms were significantly relieved.

**Figure 2:**
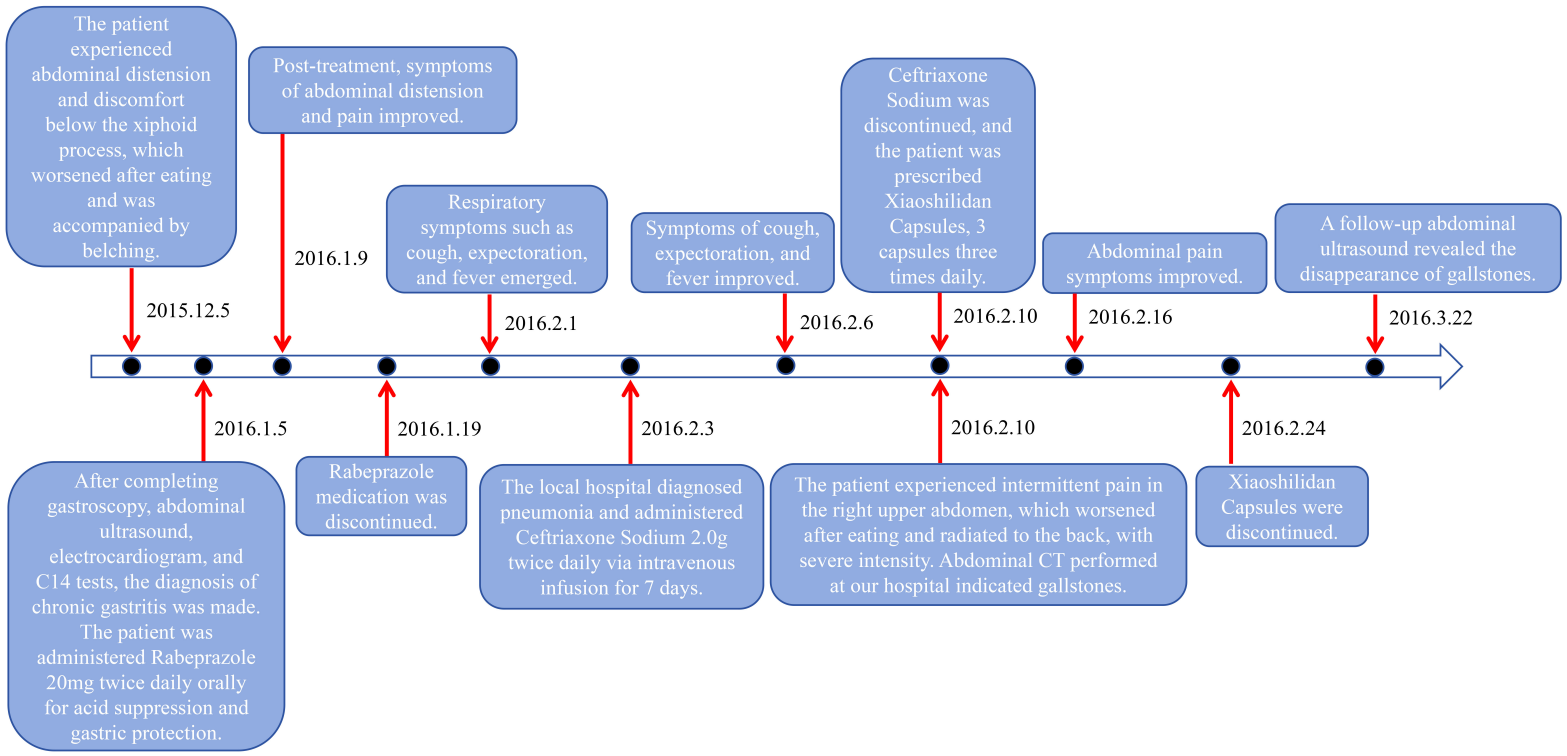
Treatment history of the case.

However, the patient developed symptoms of coughing up yellow phlegm and fever after being exposed to cold, which lasted for 2 days. Subsequently, the patient received treatment with ceftriaxone sodium at a local hospital, administered intravenously twice a day at a dose of 2.0 grams each time for a continuous period of 7 days. Nevertheless, after the treatment, the patient experienced discomfort in the right upper abdomen and thus returned to our hospital's gastroenterology department for a follow-up examination. During the follow-up physical examination, we observed that the patient's abdomen was flat and soft, with tenderness still present below the xiphoid process, and Murphy's sign was positive. To further clarify the diagnosis, we performed an upper abdominal CT scan on the patient, which revealed no thickening of the gallbladder wall but multiple high-density masses within the lumen (Figure 1B). Meanwhile, the results of liver and kidney function tests showed no significant abnormalities. Based on these findings, we tentatively diagnosed the patient with gallbladder stones, which we suspected might be related to the medication. Therefore, we decided to postpone surgical treatment and advised the patient to undergo a follow-up examination after discontinuing the medication. Six weeks after discontinuing the medication, we performed a follow-up examination on the patient (Figure 1C). The follow-up results showed that the gallbladder stone images had disappeared, further supporting our initial diagnosis that the patient's gallbladder stones were possibly reversible stones caused by the medication (Differential diagnosis: 1. True gallbladder stones: no recent use of medications prone to cause calcium salt deposition within the gallbladder. The surface is smooth and regular, with strong, sharp, and uniform posterior acoustic shadowing. The stones move relatively quickly, have a long formation time, and often persist for extended periods. 2. Inflammatory deposits in the gallbladder: typically, the echo is fine and weak, mostly without acoustic shadowing, and often accompanied by symptoms of cholecystitis such as abdominal pain and nausea. There is no recent use of medications prone to cause calcium salt deposition within the gallbladder. 3. Gallbladder sludge stones: no relevant medication history. The ultrasonographic appearance is mainly characterized by fine, strong echoes within the sac, with a loose structure. Follow-up ultrasonography observations indicate that they are not easily eliminated.). The detailed illustration of the entire treatment process is provided (Figure 2).

## 3 Discussion

### 3.1 Formation mechanism of ceftriaxone-induced pseudolithiasis

After the administration of ceftriaxone, some patients develop gallstones and urinary system stones, with the main component being ceftriaxone calcium salt. The mechanism underlying this phenomenon can be explained by the chemical structure of ceftriaxone sodium and its excretion process in the body. The chemical structure of ceftriaxone sodium includes anionic groups such as carboxylate and amide, which easily form insoluble precipitates when they come into contact with calcium ions in the human body. Ceftriaxone is primarily excreted through the liver and kidneys, with ~40 to 50% eliminated via bile and 50–60% via the kidneys. Due to the concentrating effects of the gallbladder on bile and the kidneys on urine, the concentration of ceftriaxone in the gallbladder and kidneys significantly increases (2), leading to the formation of supersaturated solutions. These supersaturated solutions further combine with calcium ions to form a large amount of insoluble ceftriaxone calcium salt precipitates. These precipitates continue to accumulate, ultimately forming stones.

The excretion pattern of ceftriaxone sodium in the body results in relatively high concentrations in bile and the kidneys (3). The anions formed after the dissociation of ceftriaxone sodium have a high affinity for calcium ions, which facilitates their binding in the bile ducts, gallbladder, and renal collecting system, forming insoluble ceftriaxone calcium salt precipitates that may potentially develop into stones (2, 4). Notably, the concentration of ceftriaxone sodium in the gallbladder can reach up to 20–150 times that in the blood. When its concentration exceeds the saturation point, it can deposit inside the gallbladder, forming a glue-like mass.

Additionally, in populations prone to stone formation, cholesterol in their bile acid pool is often highly saturated. When this population uses a large dose of ceftriaxone sodium, the concentration of the drug in bile increases and binds to bile acids, disrupting the balance of the bile acid pool, promoting cholesterol crystallization, and forming cholesterol stones. Furthermore, ceftriaxone may also increase calcium ion concentration by affecting bile acid secretion, further promoting the formation of precipitates (5). However, when patients discontinue the use of ceftriaxone sodium, its concentration in bile gradually decreases. At this point, ceftriaxone salt begins to dissolve in the bile and is gradually excreted into the intestine along with the bile. Meanwhile, the bile acid pool in the gallbladder gradually restores balance, redissolving previously formed cholesterol stones, and reducing or even eliminating deposits in the gallbladder.

### 3.2 Clinical characteristics of reversible gallbladder stones caused by ceftriaxone sodium

Most ceftriaxone-associated pseudolithiasis do not cause significant clinical symptoms, and their presence is mainly detected through ultrasonography (6, 7). Less than 20% of patients with pseudogallstones experience abdominal discomfort or pain, and some of these patients may also be accompanied by symptoms of nausea and vomiting (7). Additionally, these patients may exhibit a positive Murphy's sign reaction, and may even experience more severe clinical manifestations such as biliary colic, acute cholecystitis, or acute hepatomegaly. Meanwhile, these pathological changes may also lead to abnormal elevations in the levels of transaminase, total bilirubin, direct bilirubin, and indirect bilirubin in the serum, resulting in symptoms of jaundice (7).

Pseudolithiasis is mainly composed of granular calcium salt deposits formed by ceftriaxone sodium, mixed with trace amounts of cholesterol crystals and bilirubin components (8). These stones often exhibit irregular morphologies, and due to their short formation period and the weak binding force of the internal deposits, their structures are relatively loose. Consequently, they rarely develop into large stones that could lead to intestinal obstruction (9). In ultrasonography, pseudogallstones present as strong echo masses inside the gallbladder, with irregular shapes and rough surfaces. These echo masses may be accompanied by weak acoustic shadows or no obvious acoustic shadows, and the activity of the masses is not obvious, but they will move with changes in the patient's position. Strong echoes in the bile with no acoustic shadow behind is an ultrasonic feature of gallbladder sludge-like stones. For pseudonephrolithiasis, ultrasonography will show strong echoes accompanied by obvious acoustic shadows (6). Because ultrasonography can clearly reveal the size and number of stones, it is considered the preferred method for diagnosing pseudolithiasis. When encountering situations where the sonogram is difficult to distinguish, further discrimination can be made by combining the patient's medical history and medication records (10).

### 3.3 Characteristics of this case and lessons learned

This case involves a middle-aged patient with a previously healthy medical history who developed discomfort in the right upper abdomen after receiving a standard dose of ceftriaxone sodium for the treatment of upper respiratory tract infection. After a series of detailed examinations, including medical history review, physical examination, CT scan of the upper abdomen, and a follow-up abdominal ultrasound after discontinuation of the drug, we finally diagnosed the condition as ceftriaxone-induced reversible gallbladder stones, based on the criteria for adverse drug reactions proposed by Karch and Lasagna.

Through a comprehensive search of reports published domestically between January 1990 and August 2024 on adverse reactions of ceftriaxone sodium leading to gallstones, and after excluding literature that did not meet the criteria, a total of 54 relevant articles were collected, reporting 1,042 cases of gallstones induced by ceftriaxone sodium. All these cases were confirmed by ultrasonography or CT scans. The study found that the incidence rate was higher in male patients than in female patients, and primarily occurred in children (< 14 years old, with 172 cases) or young and middle-aged adults. The formation of gallstones mostly occurred within the treatment cycle of ceftriaxone sodium, especially within 15 days after administration, with the high-risk period being 3–7 days of continuous administration (Table 1). Excessive dosage, particularly administration exceeding the recommended dosage in the instruction manual, was identified as the main factor contributing to the onset of gallstones. It is noteworthy that these stones were relatively soft in texture and formed rapidly, and most of them disappeared spontaneously within 8–14 days after drug withdrawal without special treatment, resulting in generally good patient outcomes (Table 2). Therefore, clinicians should pay attention to the potential issue of pseudo-gallstones caused by the overuse of ceftriaxone sodium, closely monitor patients' clinical manifestations during drug administration, and immediately discontinue ceftriaxone sodium and switch to other treatment methods once pseudo-gallstones are detected.

**Table 1:** Time distribution of gallbladder stone occurrence.

**Occurrence time**	**Number of cases**
1–2 days of drug administration	6
3–7 days of drug administration	273
8–15 days of drug administration	37
Unknown^*^	478
Unknown^#^	248
Total	1,042

^*^Indicates administration for 1–15 days.

^#^Indicates no specific data given in the literature.

**Table 2:** Time to resolution of adverse reactions.

**Resolution time (days)**	**Number of cases**
1–7	51
8–14	236
15–28	68
29–42	11
43–	5
Unknown^*^	226
Unknown^#^	445
Total	1,042

^*^Indicates regression time of 1–50 days.

^#^Indicates no specific regression time in the text.

However, in this case, the patient was a middle-aged woman who developed large and multiple gallbladder stones after 7 days of ceftriaxone sodium treatment, with the largest stone measuring ~2 cm in diameter, accompanied by symptoms of upper abdominal pain. After discontinuing the medication, we provided symptomatic treatment with anti-inflammatory, cholagogue [Take 3 capsules of Xiaoshi Lidan (stone-dissolving and gallbladder-benefiting) orally, three times daily.], and analgesic therapies, and the patient's symptoms gradually improved. Notably, during a follow-up abdominal ultrasound examination 42 days after discontinuing the medication, we found that the stones had completely disappeared, thereby successfully avoiding the need for gallbladder surgery.

This case further confirms a characteristic of drug-induced stones: regardless of their size or density, they usually have the possibility of disappearing spontaneously, although the time required varies from individual to individual. This finding has important guiding significance for clinical practice and patient management.

### 3.4 Prognosis, prevention, and treatment

According to the study by Schaad et al. (11), symptoms gradually subsided and/or ultrasonography returned to normal within 2–63 days after cessation of ceftriaxone sodium in 16 children with pseudolithiasis. Another double-blind controlled study conducted by Heim-Duthoy et al. (12) showed signs of normalization observed within 9–26 days after discontinuation of ceftriaxone in patients with abnormal gallbladder ultrasonography. Additionally, Acun et al. (13) reported that the ultrasound images of right children with pseudolithiasis gradually returned to normal within 30–90 days after stopping ceftriaxone sodium, with an average time of 41 days. These findings suggest that both pseudolithiasis symptoms and abnormalities detected by ultrasonography are expected to recover naturally over a period of time after discontinuing ceftriaxone sodium.

Ceftriaxone-induced pseudolithiasis exhibits a reversible characteristic, implying that these stones often spontaneously dissolve after discontinuation of the drug, even without specific therapeutic interventions. Naturally, if patients develop other related symptoms, targeted treatment measures should be taken to alleviate them. Based on this feature, for patients diagnosed with pseudolithiasis, a more cautious observational strategy should be adopted in the absence of obvious surgical indications. Through close medical observation, we can avoid unnecessary physical suffering caused by blind surgery and alleviate patients' economic pressure.

During the treatment with ceftriaxone sodium, reasonable and standardized medication principles must be followed. It is recommended that the daily dose of the drug should not exceed 2 grams. For pediatric patients weighing < 40 kilograms, the recommended daily dose should be < 50 milligrams per kilogram of body weight. Additionally, intravenous infusion should be administered over a period of more than 30 min to ensure stable and slow entry of the drug into the body. Patients should follow the established treatment course, and for those taking medication for more than a week, regular abdominal ultrasonography and assessment of liver and kidney function should be performed. Meanwhile, special attention should be paid to fluid replacement to maintain normal body metabolism.

In pediatric medication, greater caution and close monitoring are required (14). Particular attention should be paid when administering ceftriaxone sodium to patients already suffering from cholecystitis, and concomitant use with other hepatotoxic or nephrotoxic drugs should be avoided. According to relevant studies, ceftriaxone sodium should not be mixed with calcium-containing solutions such as Hartmann's solution or Ringer's solution to prevent possible drug interactions or adverse reactions. These recommendations are based on existing medical research and clinical experience, aiming to ensure patient safety and treatment effectiveness.

## 4 Conclusion

Given the widespread clinical application of ceftriaxone sodium, there has been a gradual increase in reports of stone cases associated with its use. However, it is worth noting that most of these stones are reversible, and patients typically have a good long-term prognosis. This situation should garner widespread attention from medical practitioners. When treating patients with suspected gallstones, it is imperative to inquire about their recent history of ceftriaxone sodium use. If the patient has a history of using such medication, a follow-up observation strategy may be considered, rather than immediate surgical intervention. This approach not only helps avoid unnecessary gallbladder removal surgeries but also alleviates the economic burden on patients to some extent. Therefore, while we need to maintain high vigilance regarding the potential for ceftriaxone sodium to cause stones, there is no need for undue alarm. Through a reasonable diagnosis and treatment process, we can fully ensure the health of patients while reducing unnecessary medical interventions.

## Data Availability

The original contributions presented in the study are included in the article/Supplementary material, further inquiries can be directed to the corresponding author.
