# Supplementary material for: A case report and literature review: one case of ceftriaxone sodium-induced reversible gallbladder stone
Source: Front Med (Lausanne). 2024 Nov 27;11:1445228. doi: 10.3389/fmed.2024.1445228 (PMC11631606; doi:10.3389/fmed.2024.1445228)
Supplement: Supplementary file 2 [file Data_Sheet_2.pdf]

安康市中心医院医学伦理委员会
临床项目审批件

批件号：2024No. 025

审查日期	2024. 03. 15
临床研究项目	A case report and literature review:one case of ceftriaxone sodium-induced reversible gallbladder stone
承担科室	消化内科
主要研究者	罗长琴
伦理审查方式	□快速审查 ☑会议审查

审批意见：（一）同意
（二）做必要修改后同意
（三）不同意

伦理委员会通过对送审材料的审阅和讨论，参加投票（8）人，同意：（8）票；做必要修改后同意：（0）票；不同意：（0）票。

经 2024 年 3 月 15 日医院伦理委员会讨论研究，同意开展以上项目，要求认真总结临床经验，不断提高医疗质量，确保医疗安全。

伦理委员会主任签名：

安康市中心医院伦理委员会
2024 年 4 月 2 日
